# Increase of Antimicrobial Consumption in a Tertiary Care Hospital during the First Phase of the COVID-19 Pandemic

**DOI:** 10.3390/antibiotics10070778

**Published:** 2021-06-25

**Authors:** Alexandre Castro-Lopes, Sofia Correia, Cátia Leal, Inês Resende, Pedro Soares, Ana Azevedo, José-Artur Paiva

**Affiliations:** 1 Faculty of Medicine of the University of Porto, 4200-319 Porto, Portugal; 2 EPIUnit, Public Health Institute of University of Porto, 4050-091 Porto, Portugal; sofia.correia@ispup.up.pt (S.C.); catia.leal@ispup.up.pt (C.L.); anazev@med.up.pt (A.A.); 3 Laboratory for Integrative and Translational Research in Population Health (ITR), 4200-319 Porto, Portugal; 4 Department of Public Health and Forensic Sciences and Medical Education, Faculty of Medicine of the University of Porto, 4200-319 Porto, Portugal; 5 Pharmaceutical Service, Centro Hospitalar Universitário São João, 4200-319 Porto, Portugal; ines.resende@chsj.min-saude.pt (I.R.); pedro.soares@chsj.min-saude.pt (P.S.); 6 Hospital Epidemiology Center, Centro Hospitalar Universitário São João, 4200-319 Porto, Portugal; 7 Intensive Care Medicine Service, Centro Hospitalar Universitário São João, 4200-319 Porto, Portugal; japaiva@med.up.pt; 8 Department of Medicine, Faculty of Medicine of the University of Porto, 4200-319 Porto, Portugal; 9 Infection and Sepsis Group, Centro Hospitalar Universitário São João, 4200-319 Porto, Portugal

**Keywords:** COVID-19, drug utilization, antimicrobial agents, antimicrobial stewardship, antimicrobial consumption indicators

## Abstract

*Background*: The COVID-19 pandemic poses novel challenges in antimicrobial consumption metrics and stewardship strategies. COVID-19 patients became the major cause of hospital admission during the first wave of the pandemic, often leading to an antimicrobial prescription upon admission or treatment for superinfections. The aim of this study was to understand how antimicrobial consumption was impacted at the beginning of the pandemic in a tertiary care hospital, a reference center for COVID-19. *Materials and Methods*: A retrospective before-and-after study was done. Descriptive statistics of discharges, patient-days, and antimicrobial use indicators (defined daily doses (DDD)/100 discharges, DDD/100 patient-days) for various groups were calculated for the first three months of the pandemic (March, April, and May 2020) as a quarterly value, and for each year in 2011–2019, and their annual percentage changes were used to estimate 95% confidence intervals. The indicators were compared to patient type (medical/surgical), type of admission (urgent/elective), and age groups using Spearman’s correlation coefficient. *Results*: Statistically significant increases occurred in 2020 for total antibacterials, macrolides, cephalosporins, amoxicillin/clavulanic acid, carbapenems, meropenem, and third-generation cephalosporins, while a reduction was seen in cefazolin/cefoxitin. A correlation was found between antibacterial consumption and patient or admission type. In 2020, unlike in pre-pandemic years, there was a different impact in DDD/100 discharges and DDD/100 patient-days due to increased lengths-of-stay and longer antimicrobial therapy. *Conclusions*: The COVID-19 pandemic led to an increase in antimicrobial consumption with a different impact in DDD/100 discharges and DDD/100 patient-days. This highlights the need to use both indicators simultaneously to better understand the causes of antimicrobial consumption variation and improve the design of effective antimicrobial stewardship interventions.

## 1. Introduction

In December 2019, a novel coronavirus was identified in Wuhan, China, referred to as SARS-CoV-2. The first case of coronavirus disease 2019 (COVID-19) in Portugal was confirmed on 2 March 2020 [1], and the disease has since propagated throughout the country, with 805,647 cumulative confirmed cases and 16,389 deaths a year later [2].

Due to the surge of COVID-19 cases, from March to May 2020, elective surgical admissions were postponed, and COVID-19 became one of the major causes of hospital admissions in emergency departments, leading to a significant change in patient populations, in terms of the type of admission, diagnosis, severity, and age.

Multiple studies have shown a low incidence of bacterial or fungal coinfection in COVID-19 patients [3,4,5,6]. A review and meta-analysis by Langford et al. estimated an 8.6% risk of bacterial and fungal co-infection [7], similar to a review by Lansbury et al., which estimated a risk of 7% [8]. However, the same and other similar studies have shown frequent empirical prescriptions of antimicrobials to COVID-19 patients upon hospital admission [5,6,7,9,10,11]. Antibacterials were prescribed to between 60% [5] and 80% [11] of patients, partly due to the difficulties of differentiating isolated severe COVID-19 from COVID-19 with bacterial coinfection [12] (i.e., pneumonia due to simultaneous infection by SARS-CoV-2 and by a bacterial pathogen), as they are clinically indistinguishable before laboratory and microbiological testing, as well as due to past experience with the influenza pandemic, where bacterial coinfection was more prevalent and a major factor of poor prognosis [13,14]. The current World Health Organization (WHO) COVID-19 guidelines advise against the use of empirical antimicrobials in patients with mild or moderate COVID-19, with an emphasis on rapid de-escalation of treatment in patients with severe disease without a confirmed diagnosis of bacterial infection [15].

The local guidelines in our institution regarding the antibiotic treatment of severe community-acquired pneumonia advise on the combined use of a beta-lactam antibacterial (either amoxicillin/clavulanic acid or a third-generation cephalosporin) and a macrolide (typically azithromycin). On the other hand, although our institution has a carbapenem-sparing policy, a high incidence of extended-spectrum beta-lactamase-producing Enterobacterales and a high number of severe/critical patients with infectious syndromes requiring broad-spectrum empiric regimens determined a significant use of this class of antibiotics.

There is currently limited data on the impact of COVID-19 on antimicrobial consumption. A study conducted at the Pittsburgh VA Medical Center [16] showed a reduction in overall antibiotic days of therapy (DOT), possibly due to a decreased patient volume, evidenced by a reduction in patient-days.

The same and other studies [16,17,18,19,20] have also shown an increase in antimicrobial use per patient-day, especially azithromycin, non-antipseudomonal penicillins (such as amoxicillin and ampicillin), and several other broad-spectrum antibiotics (piperacillin/tazobactam, cefepime, vancomycin, ceftriaxone, ciprofloxacin). This could be due to antimicrobial prescriptions for COVID-19 patients in combination with the reduction of other activities, such as elective surgical procedures, which usually do not require any or, at least, extensive antimicrobial coverage. 

The impact of antibiotic overuse on antimicrobial resistance with the selection of multiresistant organisms is well known, and their emergence poses a novel threat to public health, leading to increased morbidity, mortality, and healthcare costs [21,22]. Additionally, antibiotic overuse is also associated with hospital-acquired *Clostridium Difficile* infections [23]. Portugal has a high rate of isolated multiresistant organisms—as an example, in the latest 2017 report, 14.1% of *Pseudomonas Aeruginosa* laboratory isolates presented a combined resistance to ≥ 3 antimicrobial groups (including piperacillin/tazobactam, ceftazidime, carbapenems, fluoroquinolones, and aminoglycosides) [24]. In this context, antimicrobial stewardship (AMS) programs have been and will continue to be an invaluable tool in assuring the optimal prescription of antimicrobials through education, research, and intervention [25,26]. The 2019 AWaRe (Access, Watch, and Reserve) classification was developed by the WHO to assist health care services in antimicrobial stewardship practices by dividing the current antibacterial agents into three distinct groups based on current antimicrobial resistance trends and their respective use, in order to emphasize the importance of stewardship and wise use of antibacterials in the Watch and Reserve group and increase the availability of those in the Access group [21].

The present study aims to analyze the impact of COVID-19 on antimicrobial consumption during the initial phase of the pandemic, the understanding of which could prove to be an important tool for planning antimicrobial stewardship interventions to reduce antimicrobial overuse and its effect on antimicrobial resistance and health costs [12].

## 2. Results

### 2.1. Population Characteristics

**Figure 1:**
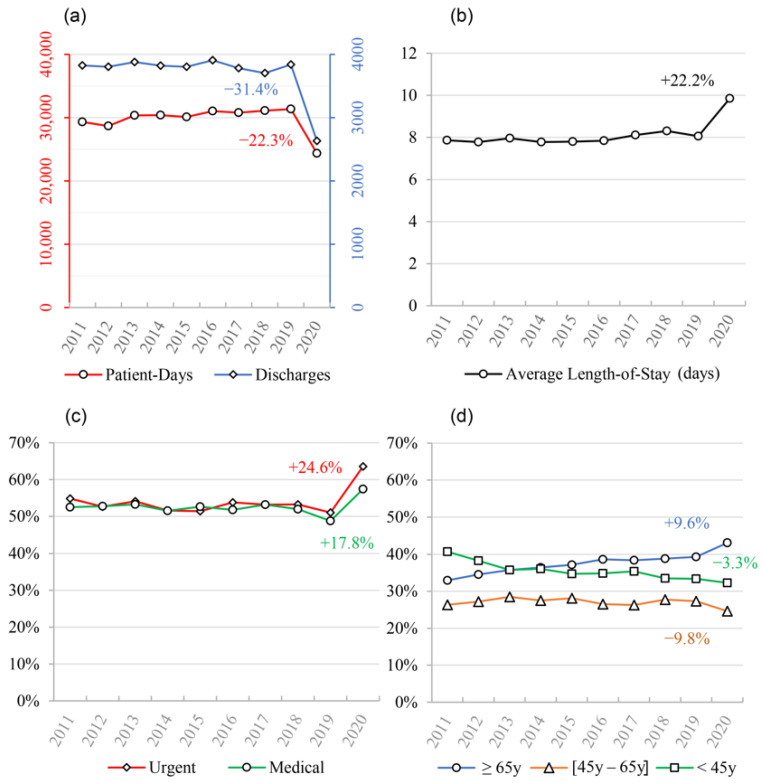
Summary of CHUSJ’s population characteristics observed between 2011 and 2020. (**a**) March–May average discharges and patient-days; (**b**) March–May average length-of-stay; (**c**) March–May average proportion of urgent and medical admissions; (**d**) March–May average proportions of age groups admitted to CHUSJ. Annual percentage changes (APCs) are shown within the graphs.

In the first 3 months of the pandemic, Centro Hospitalar Universitário de São João (CHUSJ) experienced a marked decrease in the number of discharges compared to homologous periods of previous years—we found a reduction of 31.4% in the quarterly average from 2019 to 2020. A reduction of 22.4% was also found for the average patient-days when comparing the same time periods (Figure 1a). A 22.2% increase in the average length-of-stay occurred in 2020, compared to 2019 (Figure 1b).

As expected, patient characteristics were also different between the pandemic year period and previous periods, with an increase in both the proportion of urgent admissions and medical patients when compared to 2019 (64% vs. 51%; 57% vs. 49%, respectively) (Figure 1c). A small shift in age groups admitted to the hospital was also found, with an increase in patients 65 years of age or older (Figure 1d).

### 2.2. Antimicrobial Consumption

**Figure 2:**
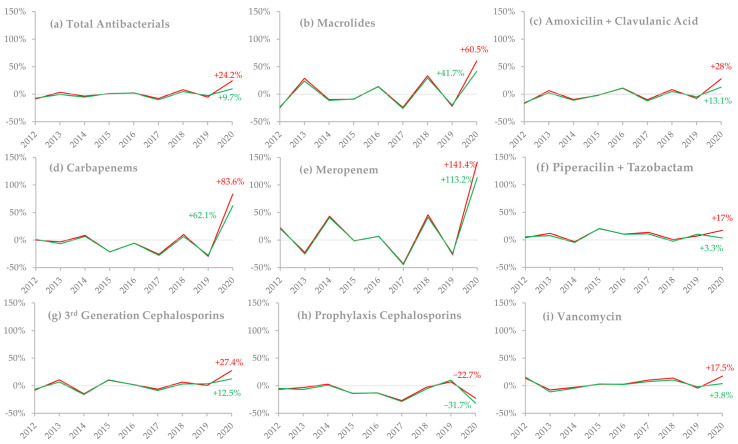
Annual percentage changes (APCs) in antimicrobial consumption between 2011 and 2020, stratified by different consumption indicators and drug groups. March–May DDD/100 discharges are represented by the red line, and March–May DDD/100 patient-days by the green line. Values for the 2020 annual percentage change are shown within the graphs. (**a**) Total antibacterials; (**b**) macrolides; (**c**) amoxicillin/clavulanic acid; (**d**) carbapenems (ertapenem/imipenem/meropenem); (**e**) meropenem; (**f**) piperacillin/tazobactam; (**g**) third-generation cephalosporins (ceftriaxone/ceftazidime/cefotaxime); (**h**) prophylaxis cephalosporins (cefazolin/cefoxitin); (**i**) vancomycin.

Annual percentage changes (APCs) from 2011–2019 were very similar for defined daily doses (DDD)/100 discharges and DDD/100 patient-days for all antibacterial subgroups when viewed on a timeline. However, in 2020, not only can we see an increase in their value compared to previous years but there was also a disparity between the values of APCs for each of the two indicators, as evidenced by their distancing and different values in this year. This occurred for several subgroups analyzed, as shown in Figure 2.

**Figure 3:**
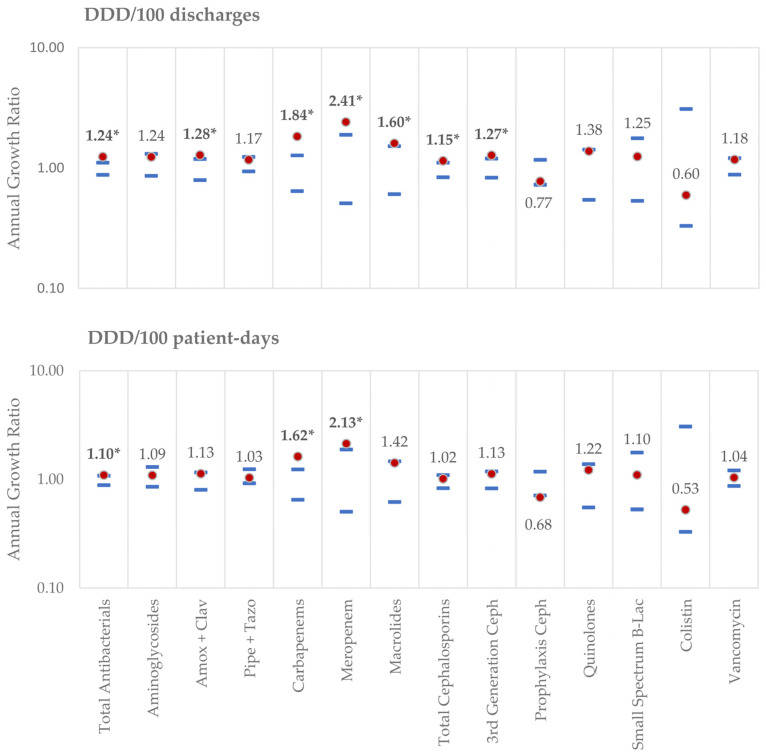
Annual growth ratio in 2020 and expected reference values for the period of 2011–2019. Calculated 2020 growth ratios (Indicatoryear (n+1)/Indicatoryear (n)) are represented by the red dot, whereas the upper and lower limits of the calculated reference values for 2011–2019 are represented by the blue lines. (*) Growth ratios outside the reference values were considered statistically significant. Growth ratios correspond to the logarithmic values of the APCs. As an example: total antibacterial use expressed as DDD/100 patient-days increased by 10% in 2020 (annual growth ratio = 1.10), whereas cefazolin/cefoxitin use decreased by 32% (annual growth ratio = 0.68).

We found statistically significant differences in antibacterial consumption in 2020 when compared to the 2011–2019 period, across several groups and subgroups, when analyzing both indicators. The findings are summarized in Figure 3.

#### 2.2.1. Assessed by DDD/100 Discharges:

There were significant increases in DDD/100 discharges in several groups: total antibacterials (1.24 vs. (0.88, 1.11)), macrolides (1.60 vs. (0.61, 1.52)), amoxicillin/clavulanic acid association (1.28 vs (0.79, 1.19)), all cephalosporins (1.15 vs. (0.84, 1.11)), third-generation cephalosporins (1.27 vs. (0.83, 1.20)), all carbapenems (1.84 vs. (0.64, 1.27)) and meropenem (2.41 vs. (0.51, 1.89)). The only groups that showed a decrease in consumption assessed by this indicator, although not statistically significant, were cefazolin/cefoxitin (0.77 vs. (0.73, 1.17)) and colistin (0.60 vs. (0.33, 3.10)).

#### 2.2.2. Assessed by DDD/100 Patient-Days:

In 2020, compared to 2019, there was a statistically significant reduction in DDD/100 patient-days for cefazolin/cefoxitin (0.68 vs. (0.71, 1.18)), whereas only total antibacterials (1.10 vs. (0.88, 1.08)), carbapenems (1.62 vs. (0.65, 1.24)) and meropenem (2.13 vs. (0.50, 1.88)) showed a statistically significant increase.

### 2.3. Extended Antimicrobial Therapy

**Figure 4:**
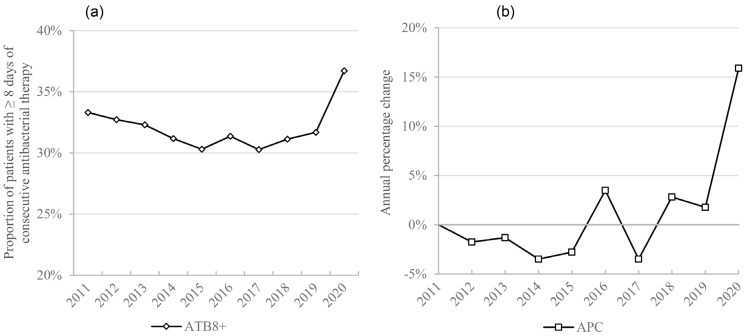
Extended antimicrobial therapy: (**a**) March–May average proportion of patients with ≥ 8 days of antibacterial therapy (ATB8+); (**b**) annual percentage change of March–May average ATB8+.

There was also a statistically significant increase in patients exposed to 8 or more days of antibacterial therapy (ATB8+) (1.16 vs. (0.94, 1.05)), increasing by 16% in 2020 when compared to 2019 (Figure 4).

### 2.4. Consumption by AWaRe Groups

**Figure 5:**
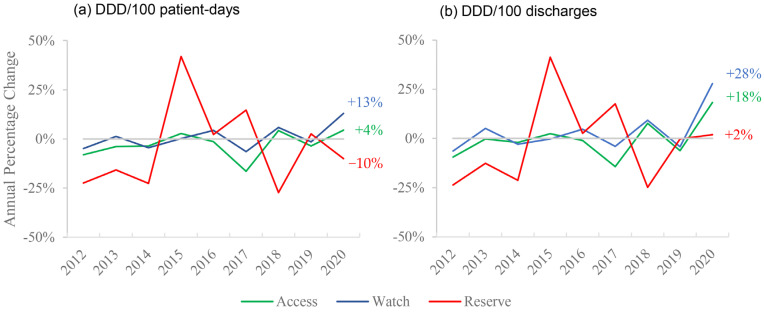
Annual percentage change of March–May consumption of different AWaRe groups: (**a**) analyzed by DDD/100 patient-days; (**b**) analyzed by DDD/100 discharges.

We observed an increase in the Access and Watch subgroups consumption during the first few months of the pandemic in CHUSJ, whereas there was a reduction or a very slight increase in the Reserve group consumption as analyzed by the DDD/100 patient-days or DDD/100 discharges, respectively, as can be seen in Figure 5.

### 2.5. Correlation Analysis

A statistically significant correlation was found between the proportion of patients aged 45–65y and antibacterial consumption when expressed as DDD/100 discharges (correlation of −0.52, *p* < 0.01), but not as DDD/100 patient-days. No other statistically significant correlation was found between the proportion of age groups and antibacterial consumption.

On the other hand, months with a higher proportion of medical patients and urgent admissions had higher antimicrobial consumption, as both the antibacterials’ DDD/100 discharges and DDD/100 patient-days correlated positively with these statistics, as seen in Table 1 (a correlation of 0.51, *p* < 0.01, and a correlation of 0.60, *p* < 0.01 for DDD/100 discharges; a correlation of 0.43, *p* = 0.019, and a correlation of 0.38, *p* = 0.039 for DDD/100 patient-days, respectively).

## 3. Discussion

During the first wave of the COVID-19 pandemic, a marked decrease in the number of patients cared for at CHUSJ was observed, evidenced by the reduction in both discharges and patient-days, which is in line with studies conducted in other hospitals [16,17,20]. There was also an increase in the average length-of-stay, probably due to an increase in the proportion of urgent admissions, medical patients, and older age groups, as well as a reduction in elective surgeries.

A substantial increase in antimicrobial use was found in various specific subgroups, either measured as DDD/100 discharges or as DDD/100 patient-days, which is consistent with other studies [16,17,19,27]. This was probably due to the fact that, on one hand, the numerator increased as patients presenting to CHUSJ were generally more severely diseased and thus, more often required antimicrobial therapy, and, on the other hand, the denominator decreased, as elective procedures accounted for the majority of the reduction in discharges and patient-days.

Some of the observed changes were expected; macrolide use increased in 2020, especially when measured as DDD/100 discharges. Azithromycin, which is responsible for 90% of macrolide administration in CHUSJ, is used as a first-line therapy against atypical community-acquired pneumonia, which has the same presentation as COVID-19, and therefore, is instituted as empirical therapy in all severe community-acquired pneumonia cases, at least until microbiological diagnosis. Azithromycin is also a singular case, as its increase could also be possibly explained by its use as an off-label treatment of COVID-19 in the first few months of the pandemic. However, that was not the case in our hospital, where its use in COVID-19 cases was merely justified by the possibility of bacterial co-infection. Similarly, third-generation cephalosporins (ceftriaxone/ceftazidime/cefotaxime) and amoxicillin/clavulanic acid, antibacterials used in lower respiratory tract infections, were also increased in their consumption in 2020, achieving a slight but nonetheless statistical significance only in DDD/100 discharges. This is once again compatible with the frequent use of antibacterials for the empirical treatment of community-acquired pneumonia, in spite of the current recommendations that advise on their wise use [15].

There was a significant increase in carbapenem use in 2020, almost completely explained by a marked increase of meropenem use of 113% in DDD/100 patient-days and 141% in DDD/100 discharges, accounting for 79% of all carbapenems prescribed in 2020, compared to an average of 43% in previous years. We may speculate that this was due to the emergence of superinfections in COVID-19 patients, often caused by multiresistant bacteria [11,28,29], and/or to a reduction in AMS activities during this period due to extreme overwork [12,30].

Cefoxitin and cefazolin, two cephalosporins administered primarily as pre-surgical prophylaxis for bacterial infections, were less used according to both indicators, achieving statistical significance in DDD/100 patient-days. This is compatible with other studies [20,31] and certainly due to the fact that on 12 March 2020, all non-urgent surgical procedures were halted (except for trauma and priority cardiac, oncologic, and vascular surgery), which reduced the amount of pre-surgical prophylaxis administered.

In previous years, APCs were very similar in both indicators (DDD/100 discharges and DDD/100 patient-days), when analyzing every subgroup of antimicrobial agents. This indicates that monitoring antimicrobial consumption trends with any of these indicators would have been similar. However, substantial differences in APCs were found between them in 2020.

Notably, in all subgroups that presented an increase in DDD/100 discharges and DDD/100 patient-days, there was a larger increase in the former, sometimes being statistically significant in DDD/100 discharges but not DDD/100 patient-days (as is the case with macrolides, cephalosporins, third-generation cephalosporins and amoxicillin/clavulanic acid). Similarly, cefazolin/cefoxitin suffered a larger reduction in DDD/100 patient-days than in DDD/100 discharges, the latter not being significant. This could be due to the fact that despite both discharges and patient-days diminishing during the pandemic, the first reduced more than the second, as patients had longer lengths-of-stay (Figure 1), which leads to the hypothesis that longer durations of antibiotic regimens were the major determinant of the increase in consumption, rather than a higher number of patients submitted to antibiotic treatment. In fact, in 2020, there was a significantly higher percentage of patients on long-duration (≥8 days) antibacterial therapy. This increase may have been due to the treatment of superinfections in COVID-19 patients, where treatment failure and the need to readapt antibacterial therapy due to antimicrobial resistance or to prevent infection relapse led to longer antibacterial courses. Additionally, patients may have had more than one hospital-acquired infection due to their increased lengths-of-stay, which may have required consecutive antibacterial treatment. On the other hand, since ATB8+ was measured as a proportion of total patients, the reduction in elective surgeries, which usually only require a single administration of antibacterial therapy, may have contributed to this increase.

Each indicator has its own use and interpretation, but the complex reality of the COVID-19 pandemic and its effect on patient types, lengths-of-stay, and admissions, among others, has impacted each one in different ways. DDD/100 patient-days is commonly the only indicator reported in antimicrobial consumption studies, as it more accurately portrays overall days of therapy, but DDD/100 discharges may be more accurate in showcasing selection pressure for resistant microorganisms [32], as it varies with the amount of antimicrobial therapy each is exposed to, independently of length-of-stay.

A significant correlation between antimicrobial consumption and the proportion of patients and types of admission was found. Generally, a higher proportion of urgent admissions and medical patients were associated with higher consumption of antibacterials. Therefore, the shift in patient types during this period admitted to CHUSJ may have contributed to our findings. Notably, we did not find a significant correlation between patient age groups and antibacterial consumption, except for a negative correlation between 45–64 y and DDD/100 discharges. The cancellation of elective surgical procedures in 2020 may have disproportionately impacted this age group without similarly affecting DDD counts (as elective surgical procedures usually require minimal antibacterial therapy), which would lead to an increase in antibacterial consumption in this indicator, mostly due to a decrease in the denominator. Additionally, different age groups may contribute to increases in certain subgroups of antibacterials and reductions in others, while not influencing the larger group of antibacterials.

We report an increase in consumption mostly in the “Access” and “Watch” groups of antibiotics from the WHO 2019 AWaRe classification, whereas the “Reserve” group suffered no increase in this period. This group accounted for only 1.13% of antibacterial consumption in CHUSJ, which explains its large variability, as small increases in consumption lead to significant changes in APC. Additionally, we can see that when analyzed by DDD/100 patient-days, there was a decrease of 10% in this group in 2020, whereas, in DDD/100 discharges, we see an increase of 2%—further emphasizing the disparity between the indicators.

However, an increase mostly in the “Watch” group is worrying, as antibacterials in this group are prone to be targets of antibiotic resistance and require close monitoring for timely stewardship interventions. Namely, azithromycin, third-generation cephalosporins, and carbapenems all belong to the “Watch” group.

AMS is an essential part of appropriate antimicrobial prescription, and the great strain imposed on healthcare services has highlighted the need for a solid foundation of stewardship strategies that will allow us to respond to future pandemics. Prospective audits and feedback, as well as preauthorization, are recognized as the most effective strategies [33], but they require a great deal of resources, which were not available during the pandemic. On the other hand, automatic stop orders and formulary restrictions are very useful in the sense that they do not require human resource intervention but may be detrimental to the communication and collaboration between AMS groups and clinicians [34]. Future studies into antimicrobial consumption and which AMS strategies were successfully implemented during the COVID-19 pandemic may help us prepare not only for future pandemics but may also provide a framework of interventions that may be used in similar resource-scarce circumstances.

Our study corroborates previous findings, where altered hospital resource indicators, such as length-of-stay and total patient-days, led to disparities between different antimicrobial use indicators [35,36]. Future studies should take into account how the choice of indicator impacts their findings and conclusions and that using more than one indicator might help to better understand the determinants of variation in antimicrobial consumption.

### Strengths and Limitations

The inclusion of multiple years preceding the pandemic in the statistical analyses allowed us to accurately estimate expected APCs in antimicrobial consumption. This was essential in evaluating which subgroups showed an increase or decrease in 2020 that were not due to either random variation or a pre-established trend, which would not have been possible if they were solely compared with 2019.

However, this was a single-center study, which may limit its applicability in other settings—a systematic review of results from different centers regarding antimicrobial consumption during the pandemic could provide additional information. Moreover, an analysis of the adequacy of prescriptions during this period would have supplied valuable data and helped to draw further conclusions.

## 4. Materials and Methods

### 4.1. Study Setting and Timing

A retrospective, descriptive before-and-after study was conducted in CHUSJ, a 1105-bed public teaching tertiary care hospital in the city of Porto, located in the north of Portugal, which was a reference center for COVID-19 during the first phase of the pandemic.

The pharmacy data of all antimicrobials dispensed for in-patients in the months of March, April, and May of each year between 2011 and 2020 were analyzed in order to provide an accurate representation of the beginning of the pandemic in Portugal and enough corresponding pre-pandemic quarters.

### 4.2. Data Extraction

The data on antimicrobial consumption was retrieved from CHUSJ’s Pharmaceutical Department database. Antimicrobials prescribed to ambulatory patients for purchase in community pharmacies, such as external consultation and discharged emergency room patients, as well as those used in day-hospital treatments, were excluded from this study.

The data on all pharmacy-dispensed antimicrobials included in the Anatomical Therapeutical Chemical (ATC) group J01 (antibacterials for systemic use) were retrieved and expressed as DDDs, according to the WHO-ATC/DDD index [37]. 

The analyzed groups and subgroups were defined as follows: total antibacterials, aminoglycosides, macrolides, “small-spectrum” beta-lactams (penicillin/ampicillin/flucloxacillin/amoxicillin), piperacillin/tazobactam, amoxicillin/clavulanic acid, quinolones, carbapenems (ertapenem/imipenem/meropenem; meropenem was additionally analyzed by itself, as it showed a large increase not seen in the other carbapenems), cephalosporins, cephalosporins used for surgical prophylaxis (cefoxitin/cefazolin), third-generation cephalosporins (ceftriaxone/cefotaxime/ceftazidime), vancomycin, and colistin (colistimethate sodium).

The percentage of patients with 8 or more consecutive days of antibacterial therapy (ATB8+) was also retrieved on a monthly basis and expressed as a quarterly average for each year. Antibacterials were also divided into subgroups and analyzed based on the 2019 WHO AWaRe classification.

To further characterize the hospitalized population, administrative data was retrieved, which included the number of discharges, hospitalization days (patient-days), average length-of-stay, type of admission (elective vs. urgent), type of patients according to the diagnosis-related group (DRG) (medical vs. surgical) and patients’ ages, aggregated into the following age groups: < 45; 45–64; ≥ 65 (< 18 and (18–45) classes were aggregated into < 45, and (65–75) and ≥ 75 classes into ≥ 65, as they showed similar results).

All data were retrieved from the hospital’s administrative database through an in-house business intelligence platform (HVITAL), described elsewhere [38].

### 4.3. Statistical Analysis

Global antimicrobial consumption and specific subgroup consumptions were calculated and expressed as DDD/100 patient-days and DDD/100 discharges [39,40]. Since antimicrobial use in March, April, or May within each year was very similar, the indicators were analyzed as a quarterly value for each year within each subgroup, using the following formulas:

(1) $$DDD/100\;discharges\;\left(year\right)=\frac{Sum\;DDDs_{\left(March+April+May\right)}}{Sum\;discharges_{\left(March+April+May\right)}}*100$$

(2) $$DDD/100\;patient\;days\;\left(year\right)=\frac{Sum\;DDDs_{\left(March+April+May\right)}}{Sum\;patient\;days_{\left(March+April+May\right)}}*100$$

For each indicator and subgroup, the corresponding APC was estimated on a yearly basis, as follows:

(3) $${\mathrm{APC}}_{\mathrm{year}\left(n+1\right)}=\frac{{\mathrm{Indicator}}_{\mathrm{year}\;\left(n+1\right)}-{\;\mathrm{Indicator}}_{\mathrm{year}\left(n\right)}\;}{{\mathrm{Indicator}}_{\mathrm{year}\left(n\right)}}*100\%$$

To assess if the 2020 annual variation was significantly different from what was expected, it was compared with the pattern observed in previous years; the annual growth ratio (*Indicatoryear (n+1)/Indicatoryear (n)*) was calculated to assess the reference values for the period of 2011–2019 that were estimated as the 95% confidence intervals of the annual growth ratio. The 2020 annual variation was considered significantly different if the growth ratio was above the calculated upper reference value or below the calculated lower reference value. Similar calculations were also done for ATB8+.

Population characteristics were described as monthly values (number of discharges, hospitalization days, and average length-of-stay) and proportions (type of admission, type of DGR-related patients, and patients’ age groups), and later calculated for each year on a quarterly basis, for descriptive analysis.

Each indicator’s monthly data were also used for correlation analysis. Spearman’s correlation coefficient was calculated to evaluate the relation between monthly total antimicrobial consumption and the previously mentioned population characteristics, for the whole duration of the study (2011–2020).

A *p*-value < 0.05 was considered statistically significant. All data analysis and calculations were done using Microsoft Excel 2019 (Microsoft Corporation, Redmond, WA, USA) and R software (The R Foundation for Statistical Computing, 1020 Vienna, Austria) version 4.0.35.

## 5. Conclusions

In the 3 months of the first wave of the COVID-19 pandemic, a worrying increase in antibacterial consumption occurred but was observed to be different between the considered indicators. Such an increase seems to result from a marked change in the number and characteristics of hospitalized patients and reflects longer durations of antibiotic courses, as opposed to a higher rate of patients treated with antibiotics.

Future studies on antimicrobial use during COVID-19 and future pandemics should consider that the use of multiple indicators and an exploration of hospital resource indicators is necessary to analyze the determinants of variation, accurately draw conclusions, and plan antimicrobial stewardship interventions.

## Figures and Tables

**Table 1:** Correlation coefficients between population characteristics and total antibacterial consumption.

Patients	DDD/100 Discharges	DDD/100 Patient-Days
Urgent Admissions	**0.6 (<0.001)**	**0.38 (0.039)**
Medical Patients	**0.51 (0.004)**	**0.43 (0.019)**
<45y	−0.1 (0.600)	0.3 (0.110)
45–64y	**−0.52 (0.004)**	−0.28 (0.130)
≥65y	0.19 (0.310)	−0.23 (0.210)

Statistically significant correlations are in bold.

## Data Availability

All data presented in this study are available on request by contacting the corresponding author.
